# Targeting erythroid cell–derived asparagine inhibits alloimmunization in sickle cell disease^∗^

**DOI:** 10.1182/bloodadvances.2026020233

**Published:** 2026-04-20

**Authors:** Kai Dou, Shan Su, Weili Bao, Yunfeng Liu, Qiao Jin, Deepa Manwani, Irina Murakhovskaya, Sally Campbell-Lee, Patricia Shi, Cheryl Lobo, Xiuli An, Karina Yazdanbakhsh, Hui Zhong

**Affiliations:** 1 Laboratory of Immune Regulation, Lindsley F. Kimball Research Institute, New York Blood Center, New York, NY; 2 Laboratory of Complement Biology, Lindsley F. Kimball Research Institute, New York Blood Center, New York, NY; 3 Division of Pediatric Hematology-Oncology, Department of Pediatrics, Albert Einstein College of Medicine, Children's Hospital at Montefiore, Bronx, NY; 4 Department of Hematology and Oncology, Albert Einstein College of Medicine/Montefiore Medical Center, Bronx, NY; 5 Department of Pathology, University of Illinois at Chicago, Chicago, IL; 6 Clinical Research in Sickle Cell Disease, Lindsley F. Kimball Research Institute, New York Blood Center, New York, NY; 7 Laboratory of Blood-Borne Parasites, Lindsley F. Kimball Research Institute, New York Blood Center, New York, NY; 8 Laboratory of Membrane Biology, Lindsley F. Kimball Research Institute, New York Blood Center, New York, NY

## Abstract

Red blood cell (RBC) transfusions remain a life-saving therapy for patients with sickle cell disease (SCD), yet their safety and efficacy are limited by a high incidence of alloimmunization. Emerging evidence suggests that metabolites, including amino acids, can influence humoral immune responses. Here, we found that levels of l-asparagine (Asn), a metabolite previously implicated in immunoregulation, are increased in both plasma and erythroid cells in SCD mice. Reciprocal transfusion indicated that circulating erythroid cells contribute to plasma Asn levels, as transfusion of normal RBCs into SCD mice reduced plasma Asn, whereas transfusion of SCD erythroid cells into wild-type mice increased plasma Asn. We further observed that increased mitochondria-positive (Mito^+^) erythroid cells in SCD were associated with enhanced Asn synthesis. Functionally, depletion of Asn using asparaginase (ASNase) reduced RBC alloimmunization in SCD mice, albeit more strongly than in control mice, whereas exogenous Asn administration increased alloimmunization in wild-type mice. In SCD mice, treatment with ASNase was associated with inhibition of plasma cell differentiation while sparing resting B cells. In vitro studies revealed that ASNase inhibited, whereas Asn supplementation promoted, human B-cell differentiation, with a more pronounced effect in cells from patients with SCD. ASNase treatment was also associated with reducing Src family kinase (SFK) activation in SCD, suggesting a potential link between Asn and SCD B-cell signaling. Together, these findings point to a potential role for Mito^+^ erythroid cell Asn-SFK axis in regulating RBC alloimmunization in SCD. Modulation of this pathway may provide a basis for future therapeutic exploration.

## Key Points

• Mito^+^ circulating erythroid cells contribute to elevated plasma Asn levels in SCD mice.
• ASNase inhibits plasma B-cell differentiation more profoundly in SCD, partly by inhibiting SFK activation in activated B cells.

## Visual Abstract

**Figure:**
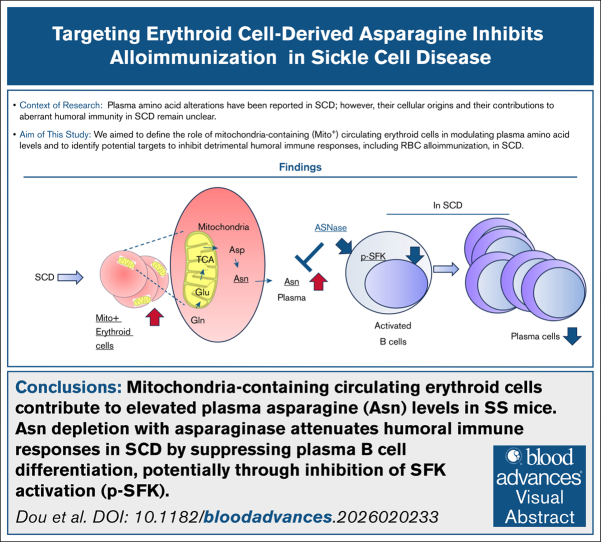

## Introduction

Sickle cell disease (SCD) is caused by a point mutation in the β-globin gene that results in structurally abnormal hemoglobin, leading to chronic hemolysis and anemia.^1^ Despite recent advances in disease-modifying therapies, red blood cell (RBC) transfusion remains a cornerstone treatment for both acute and chronic complications of SCD.^2^^,^^3^ However, transfusion therapy is frequently complicated by the development of RBC alloantibodies, which can lead to delayed hemolytic transfusion reactions and significantly limit the availability of compatible blood units.^3^ Currently, there are no approved therapies to prevent RBC alloimmunization, and the underlying mechanisms remain incompletely understood.^3^, ^4^, ^5^

Emerging evidence indicates that metabolic cues, including amino acids, play critical roles in regulating immune responses.^6^^,^^7^ Beyond serving as substrates for protein synthesis, amino acids contribute to immune cell activation and differentiation by supporting metabolic reprogramming and, in some cases, acting as signaling molecules.^8^ Notably, asparagine (Asn) has been found to directly modulate immune signaling pathways, including enhancing CD8^+^ T-cell activation through interaction with Lck, a member of the Src family kinases (SFKs).^9^ In parallel, altered amino acid metabolism has been reported in patients with SCD, particularly during vaso-occlusive crises (VOC), suggesting a potential link between metabolic dysregulation and immune function in this disease.^10^, ^11^, ^12^, ^13^ However, whether specific amino acids contribute to the risk of alloimmunization in SCD remains unexplored.

The mechanisms driving altered amino acid metabolism in SCD are not well defined. Circulating erythroid cells, including both mature RBCs and reticulocytes, are the most abundant cellular component of the blood. Several mechanisms have been identified that regulate erythroid cell metabolism, including hemoglobin-mediated control of the balance between glycolysis and the pentose phosphate pathways through binding to band 3 protein.^14^, ^15^, ^16^ However, circulating erythroid cells have traditionally been viewed as metabolically limited due to the absence of organelles in mature RBCs.^17^ In contrast, reticulocytes retain mitochondria and other organelles, enabling active metabolic processes, including amino acid synthesis.^18^ Importantly, recent studies have demonstrated abnormal mitochondrial retention in circulating erythroid cells in SCD, suggesting a potential alteration in erythroid cell metabolism.^19^, ^20^, ^21^ Whether this mitochondrial retention contributes to systemic metabolic changes, including altered plasma amino acid levels, remains unclear.

In this study, we investigated the role of circulating erythroid cells in shaping plasma amino acid profiles in SCD and evaluated how these metabolic alterations influence humoral immune responses. Our findings support that the mitochondria in SCD erythroid cells enhance Asn synthesis and that erythroid cell–derived Asn contributes to B-cell–mediated immunity. Functionally, modulation of Asn levels alters plasma cell differentiation and RBC alloimmunization, potentially through regulation of SFK signaling in SCD. These findings reveal a previously unrecognized erythroid cell–metabolism–immune axis and suggest potential therapeutic strategies to prevent alloimmunization in SCD.

## Materials and methods

Detailed experimental procedures are described in the supplemental Data. Briefly, plasma and RBC samples were collected from SCD mice (SS mice), control mice (AA mice), healthy donors (HDs), and patients with SCD without recent RBC transfusion for metabolomic analysis. Mice were immunized with RBCs from HuGPA-Tg mice (transgenic mice expressing human glycophorin A on RBCs) or with NP-CGG (4-hydroxy-3-nitrophenylacetyl–conjugated chicken gamma globulin), with or without treatment with asparaginase (ASNase), Asn, or SFK inhibitors. Antigen-specific immunoglobulin G (IgG) levels and splenic plasma cell frequencies were analyzed by flow cytometry. Naive B cells were purified from the peripheral blood of HDs and patients with SCD on a chronic transfusion program and stimulated in vitro in the presence or absence of ASNase or Asn for 5 days. B-cell survival, proliferation, plasma cell differentiation, and SFK activation were analyzed by flow cytometry.

This study was conducted in accordance with the Declaration of Helsinki. All studies were approved by the Institutional Review Board of the New York Blood Center, Montefiore Medical Center, and the University of Illinois at Chicago. All animal experiments were approved by the Institutional Animal Care and Use Committee of the New York Blood Center.

## Results

### Circulating erythroid cells contribute to the elevated levels of plasma Asn and glycine in SS mice

**Figure 1.**
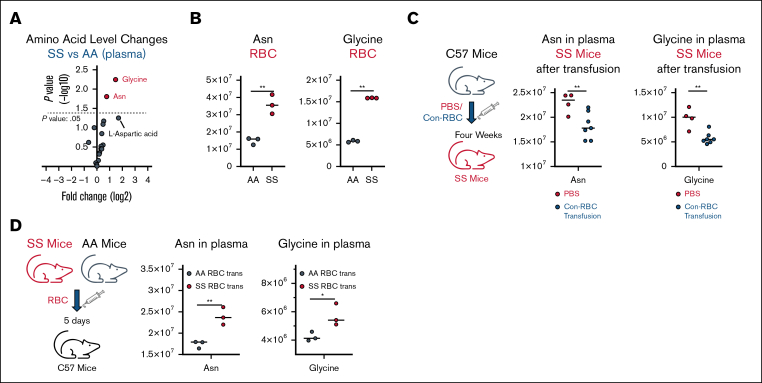
**Circulating erythroid cells contribute to elevated plasma Asn and glycine levels in SS mice.** (A) The levels of 20 protein synthesis–associated amino acids in the plasma were assessed using targeted metabolomics and compared between AA and SS mice (3 mice per group). Fold changes (log_2_) and *P* values (-log_10_) were calculated and demonstrated with the volcano plot. *P* < .05 was used as the significance threshold. The metabolites that have changed significantly are labeled in red. (B) The levels of Asn and glycine in RBCs were assessed using targeted metabolomics and compared between AA and SS mice (3 mice per group). (C) SS mice were transfused with RBCs from C57 mice (n = 7) or the same volume of PBS (n = 4) for 4 weeks, and Asn and glycine levels in the plasma were measured by metabolomic analysis. (D) RBCs from SS mice or AA mice were transfused into C57 mice (n = 3 per group), and Asn and glycine levels in the plasma were analyzed after 5 days. Circulating erythroid cells are referred to as RBCs in the figures and figure legends. ∗*P* < .05; ∗∗*P* < .01.

To identify alterations in plasma amino acid profiles in SCD, we performed targeted metabolomic analysis comparing SS and AA mice. Among 20 proteinogenic amino acids analyzed, Asn (approximately twofold) and glycine (approximately threefold) were significantly increased in SS mice (Figure 1A). Given that circulating erythroid cells, including mature RBCs and reticulocytes, are the most abundant cellular component in the blood, we next evaluated whether these cells contribute to elevated plasma amino acid levels. Metabolomic analysis of circulating erythroid cells revealed increased levels of both Asn and glycine in SS mice compared with AA controls (Figure 1B). To directly assess the contribution of erythroid cells, we performed reciprocal transfusion experiments. Transfusion of circulating erythroid cells in SS mice with RBCs from C57BL/6J mice (C57 mice, 800 μL packed cells per week for 4 weeks) reduced reticulocyte frequency to near baseline levels, confirming suppression of endogenous erythropoiesis (supplemental Figure 1). This intervention significantly decreased plasma Asn and glycine levels compared with phosphate-buffered saline–treated controls (Figure 1C). Conversely, transfusion of circulating erythroid cells from SS mice to C57 recipients (400 μL on days 1 and 3, with analysis on day 5) resulted in increased plasma Asn and glycine levels compared with mice receiving AA erythroid cells (Figure 1D).

Together, these findings suggest that circulating erythroid cells contribute to elevated plasma Asn and glycine in SCD mice. Given that previous studies have highlighted the immunoregulatory effects of Asn^9^^,^^22^ and the availability of ASNase as a drug for in-depth study,^23^ we focused on the role of Asn in this study.

### Mitochondria-containing erythroid cells play a role in Asn synthesis in SCD

**Figure 2.**
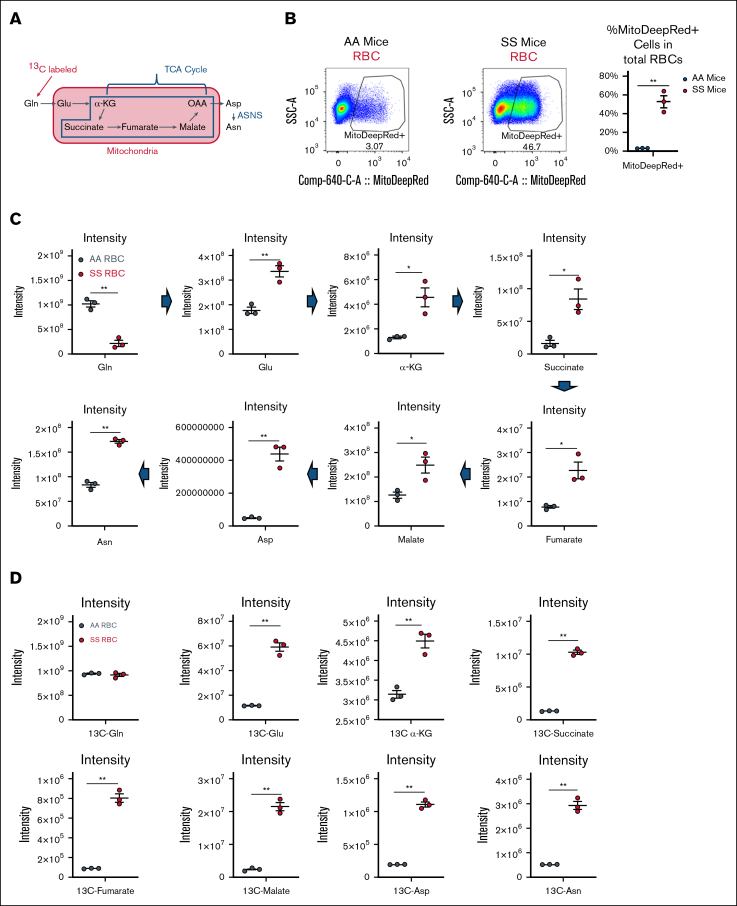

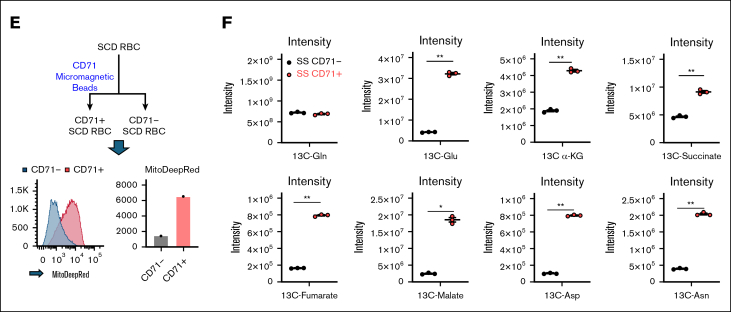
**Mitochondria-containing erythroid cells contribute to Asn synthesis in SCD.** (A) The diagram illustrates the pathway for the synthesis of Asn from Gln. (B) The frequencies of Mito^+^ RBCs in AA mice (n = 3) and SS mice (n = 3) were assessed using flow cytometry. (C) Levels of metabolites in the Asn synthesis pathway in RBCs were compared between AA mice and SS mice (n = 3). (D) RBCs from AA or SS mice (n = 3) were cultured in ^13^C-labeled Gln-supplemented and Asn-free culture medium. ^13^C-labeled metabolite levels in the Asn synthesis pathway in the culture medium were compared. (E) CD71^+^ and CD71^−^ SCD RBCs were isolated from total SCD RBCs using biotin-labeled anti-CD71 antibody and anti-biotin magnetic microbeads. The mitochondrial content in purified CD71^+^ and CD71^−^ SCD RBCs was assessed by flow cytometry. (F) Purified CD71^+^ and CD71^−^ SCD RBCs were cultured in ^13^C-labeled Gln-supplemented and Asn-free culture medium. The levels of the metabolites in the Asn synthesis pathway in the culture medium were compared. Representative data from 3 separate experiments. Circulating erythroid cells are referred to as RBCs in the figures and figure legends. ∗*P* < .05; ∗∗*P* < .01. α-KG, alpha-ketoglutarate; ASNS, asparagine synthetase; OAA, oxaloacetate.

Elevated Asn levels in circulating erythroid cells may result from increased intracellular synthesis or altered transport. As Asn can be synthesized from glutamine (Gln) through a tricarboxylic acid cycle and mitochondrial-dependent pathway (Gln-Asn pathway; Figure 2A),^24^^,^^25^ we investigated whether mitochondrial retention contributes to enhanced Asn production in SCD erythroid cells.

Using MitoTracker Deep Red staining, we observed a marked increase in mitochondria-positive (Mito^+^) erythroid cells in SS mice (46.7%) compared with AA mice (3.1%) (Figure 2B; supplemental Figure 2), consistent with a previous report^20^ and SCD-associated compensatory reticulocytosis. Metabolomic analysis further revealed alterations in multiple intermediates within the Gln-Asn synthesis pathway in SS erythroid cells relative to AA controls (Figure 2C; oxaloacetate is not detected in all samples).

To directly assess Asn synthesis, we performed stable isotope tracing using ^13^C-labeled Gln in Asn-free culture medium. Newly synthesized ^13^C-labeled intermediates in the Asn synthesis pathway were significantly increased in cultures of SS erythroid cells compared with equal volumes of AA controls (Figure 2D), indicating enhanced Asn production rather than impaired transport. To determine whether Mito^+^ erythroid cells are responsible for this effect, we enriched CD71^+^ erythroid cells from SS mice, which exhibited higher mitochondrial content compared with CD71^−^ cells (Figure 2E). When equal volumes of CD71^+^ and CD71^−^ erythroid cells were cultured with ^13^C-labeled Gln, the CD71^+^ fraction generated significantly higher levels of labeled metabolites in the Asn synthesis pathway (Figure 2F), indicating increased Asn synthesis capacity in mitochondria-enriched erythroid cells.

### Asn depletion suppresses humoral immune responses and RBC alloimmunization in SS mice

**Figure 3.**
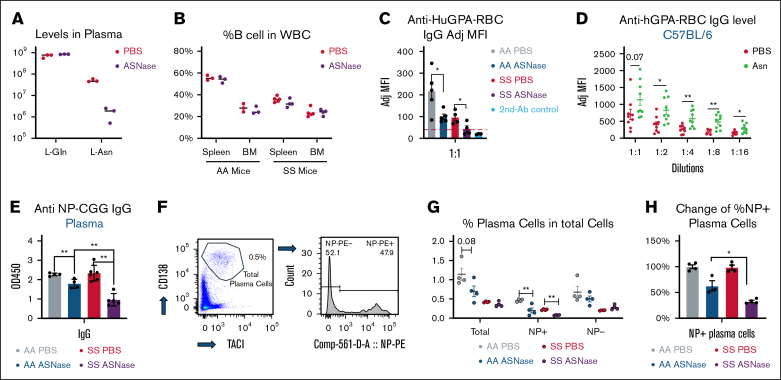
**Asn depletion inhibits humoral immune responses and RBC alloimmunization in SS mice.** SS mice were treated with ASNase or PBS (3 mice per group) for 1 week, after which (A) plasma Gln and Asn levels were measured by metabolomic analysis, and (B) B-cell frequencies in the spleen and BM were assessed by flow cytometry. (C) AA (n = 10, 5 mice per group) and SS mice (n = 8, 4 mice per group) with or without ASNase treatment were immunized with HuGPA-RBCs, and anti–HuGPA-RBC IgG levels in the plasma were assessed by the indirect antiglobulin test using flow cytometry. The adj MFI is illustrated in the column figure. (D) C57 mice with or without Asn treatment (9 mice per group) were immunized with HuGPA-RBCs, and anti–HuGPA-RBC IgG levels in the serially diluted plasma were assessed. (E) AA (n = 8) and SS mice (n = 10) were immunized with NP-CGG with/without ASNase treatment, and anti–NP-CGG IgG levels in the plasma were assessed by ELISA. (F) The pseudocolor plots reveal the gating strategy for the analysis of total and NP^+^ and NP^–^ plasma cells. (G) The frequency of total and NP^+^ and NP^–^ plasma cells in splenic CD45^+^ cells of mice (4 mice per group) in panel E was assessed by flow cytometry. (H) Fold change in the frequencies of NP^+^ plasma cells in panel G was calculated. ∗*P* < .05; ∗∗*P* < .01. Adj MFI, adjusted mean fluorescence intensities; BM, bone marrow; ELISA, enzyme-linked immunosorbent assay; NP, 4-hydroxy-3-nitrophenylacetyl; WBC, white blood cell.

To determine the functional role of Asn in humoral immunity, we first assessed the effect of ASNase on plasma Asn levels.^26^ Administration of ASNase (2.5 U per mouse, twice weekly), with a dose often used in previous studies,^24^^,^^27^ resulted in an ∼20-fold reduction in plasma Asn levels. Notably, ASNase treatment did not reduce levels of Gln (Figure 3A), the most well-known potential off-target of ASNase,^28^^,^^29^ or any other measured metabolites (data not revealed), indicating effective and selective depletion of Asn. At this dose, ASNase treatment did not alter total B-cell frequencies in either AA or SS mice (Figure 3B), suggesting minimal effects on resting B-cell homeostasis.

We next evaluated the impact of Asn depletion on RBC alloimmunization. AA and SS mice were transfused with RBCs from HuGPA-Tg mice (100 μL per mouse, receiving 2 transfusions 1 week apart, CpG was used as adjuvant for the first immunization) in the presence or absence of ASNase treatment (2.5 U per mouse, IV, twice per week, starting 1 week before immunization and continued until mice were euthanized). ASNase significantly reduced anti–HuGPA-RBC (red blood cells with human GPA expression) IgG (targeting HuGPA antigen and potentially other FVB alloantigens on donor RBCs) responses in both AA and SS mice (Figure 3C). Consistent with a previous report, SS mice displayed an overall lower alloimmunization response compared with AA controls.^30^ ASNase treatment effectively suppressed alloimmunization, with a markedly stronger effect in SS mice; notably, ∼50% of the ASNase-treated animals exhibited antibody levels below the threshold of positivity. To determine whether increased Asn levels enhance humoral responses, we supplemented C57 mice with exogenous Asn (25 μg per mouse, intraperitoneally [IP], twice per week, started 1 week before immunization and continued until mice were euthanized). Asn administration significantly increased anti–HuGPA-RBC IgG responses after RBC transfusion (Figure 3D), supporting a positive role for Asn in promoting alloimmunization.

We next evaluated whether ASNase affects antigen-specific plasma cell responses using an NP-CGG immunization model with Alhydrogel as the adjuvant. ASNase treatment reduced NP-specific IgG levels in both AA and SS mice, with a greater degree of inhibition observed in SS mice (Figure 3E). Flow cytometric analysis revealed that ASNase selectively reduced the frequency of NP-positive (NP^+^) plasma cells, while having minimal effect on NP-negative (NP^−^) plasma cells (Figure 3F-G; supplemental Figure 3), indicating that ASNase primarily inhibits ongoing antigen-driven plasma cell differentiation rather than targeting preexisting plasma cells. Consistent with the RBC alloimmunization model, ASNase-mediated suppression of NP^+^ plasma cells was more pronounced in SS mice (Figure 3H).

Together, these findings demonstrate that Asn promotes humoral immune responses and that its depletion by ASNase is associated with suppression of plasma cell differentiation and RBC alloimmunization, which is more pronounced in the SCD setting.

### Asn regulates human plasma B-cell differentiation and exhibits enhanced effects in SCD

**Figure 4.**
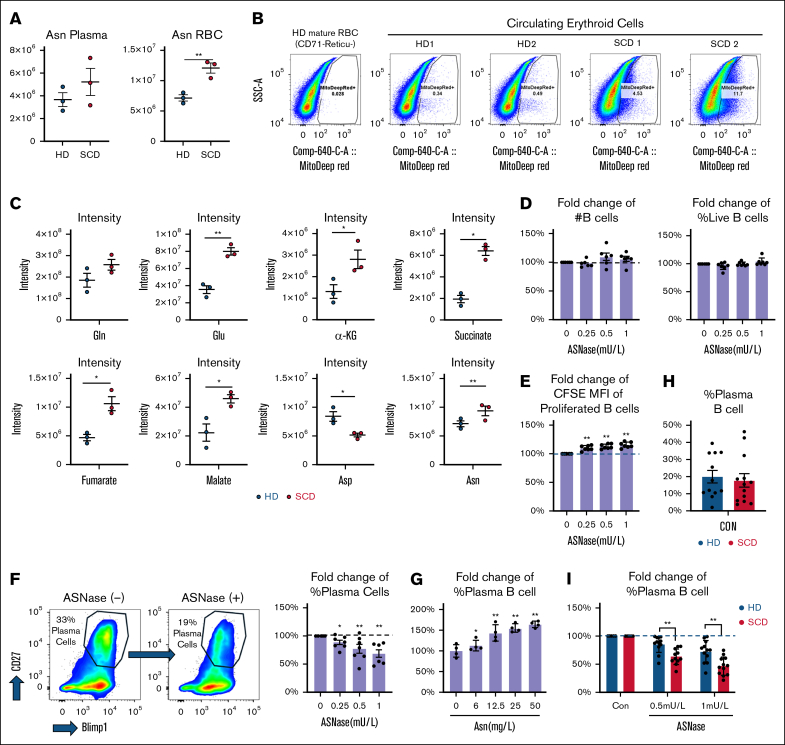
**Asn regulates human plasma B-cell differentiation and shows enhanced effects in SCD.** (A) Asn levels in the plasma and RBCs from HDs and patients with SCD (n = 3) were analyzed by targeted metabolomics. (B) The frequency of the Mito^+^ RBCs from HD and patients with SCD was assessed using flow cytometry. MitoDeepRed^+^ gate was established using staining results from HD’s mature RBCs (CD71^-^Reticu^-^). (C) Levels of metabolites in the Asn synthesis pathway (Figure 2A) in circulating erythroid cells were compared between HDs and patients with SCD (n = 3). Human naive B cells (n = 7) were isolated from the peripheral blood, stained with CFSE, and cultured in the presence of different concentrations of ASNase for 5 days. (D) Fold change in total B-cell number and frequencies of live B cells after culture were calculated (n = 7). (E) Fold change in MFI of CFSE in proliferated B cells was calculated (high CFSE MFI indicates low cell proliferation). (F) The pseudocolor plots illustrate the percentages of the plasma cells in proliferated B cells. The graph illustrates the fold change in plasma cell frequencies in response to different concentrations of ASNase (n = 7). (G) Human naive B cells (n = 4) were isolated from the peripheral blood, stained with CFSE, and cultured in the presence of different concentrations of Asn for 5 days. Fold changes in plasma cell frequency in response to different concentrations of Asn (n = 4) were calculated. (H) Human naive B cells from HDs (n = 12) and patients with SCD (n = 13) were cultured as described previously. Plasma cell frequencies in proliferated B cells were compared between HDs and patients with SCD at baseline. (I) Human naive B cells were cultured as described in panel H in the presence of different concentrations of ASNase. Fold changes in plasma cell frequency in proliferated B cells were calculated and compared between HDs and patients with SCD. Circulating erythroid cells are referred to as RBCs in the figures and figure legends. ∗*P* < .05; ∗∗*P* < .01. MFI, mean fluorescence intensities.

We next evaluated Asn levels in the plasma and circulating erythroid cells from patients with SCD and HDs. Asn levels were elevated in erythroid cells from patients with SCD, whereas no consistent difference was observed in the plasma (Figure 4A). Consistent with findings in mice, patients with SCD exhibited higher frequencies of circulating Mito^+^ erythroid cells compared with HDs, as assessed by MitoTracker Deep Red staining (Figure 4B; supplemental Figure 2), consistent with prior report^31^ and the characteristic reticulocytosis. Metabolomic analysis further revealed increased levels of intermediates in the Asn synthesis pathway in SCD erythroid cells relative to controls (Figure 4C), supporting enhanced Asn production in human circulating SCD erythroid cells.

Our data suggest that ASNase targets plasma B-cell differentiation in SS mice (Figure 3). To determine the functional impact of Asn on human B cells, naive B cells from HDs were cultured under plasma cell differentiation conditions in the presence of ASNase or Asn supplementation. Briefly, naive B cells, stained with carboxyfluorescein diacetate succinimidyl ester (CFSE) and stimulated with a B-cell activation cocktail,^32^ are cultured for 5 days, and then the frequency of plasma cells (CD27^hi^Blimp1^+^) within CFSE-low proliferated cells is measured (supplemental Figure 4). ASNase treatment (1 mU/L) selectively depleted Asn in culture medium^9^ without affecting B-cell viability, as indicated by no observed changes in total B-cell counts or viability (frequency of live cells) after culture (Figure 4D). Asn depletion reduced B-cell proliferation, as indicated by increased CFSE intensity (Figure 4E), and significantly inhibited plasma cell differentiation (Figure 4F, fold change calculated; individual data in supplemental Figure 5). Conversely, using Asn-free RPMI 1640 medium supplemented with different doses of Asn and 10% dialyzed fetal bovine serum (dialyzed with molecular weight [MW] 10 000 Da cutoff membrane to decrease small molecule levels), Asn supplementation promoted plasma cell differentiation in a dose-dependent manner (Figure 4G).

We next compared the effects of ASNase on B cells from HDs and patients with SCD. Baseline plasma cell differentiation was comparable between groups (Figure 4H). However, ASNase-mediated inhibition of plasma cell differentiation was significantly greater in B cells from patients with SCD than in HDs (Figure 4I).

Together, these findings demonstrate that Asn regulates human plasma B-cell differentiation and that B cells from patients with SCD exhibit increased sensitivity to Asn depletion.

### Asn depletion is associated with inhibition of SFK activation in SCD B cells

Given that Asn has been reported to regulate SFK signaling in T cells^9^ and SFK activation is key to both B-cell differentiation and immune function,^33^, ^34^, ^35^ we investigated whether Asn depletion modulates SFK activation in B cells.

**Figure 5.**
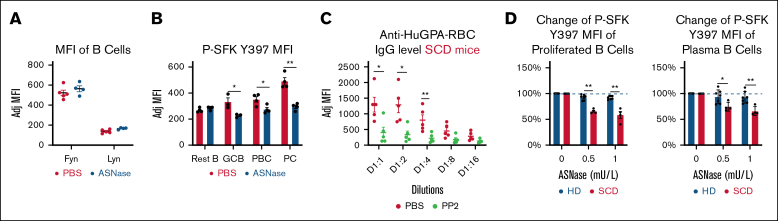
**Asn depletion is associated with inhibition of SFK activation in B cells from SCD patients. SS mice were immunized with NP-CGG as described in**Figure 3**in the presence and absence of ASNase (4 mice per group).** (A) Expression levels of Fyn and Lyn in total splenic B cells from immunized mice (4 mice per group) were analyzed by flow cytometry and illustrated in adj MFI. (B) Levels of P-SFK Y397 in splenic rest B cells, GCB, PBC, and PC from immunized mice (4 mice per group) were measured by flow cytometry and illustrated in adj MFI. (C) SS mice were immunized with HuGPA-RBCs as described in Figure 3 in the presence and absence of SFK inhibitor (PP2, 5 mice per group). Anti–HuGPA-RBC IgG levels in the plasma from PBS-treated or PP2-treated SS mice (5 mice per group) were measured with a double-dilution method and analyzed by flow cytometry. Adj MFIs are illustrated in the dot plot. (D) Naive B cells from HDs (n = 7) and patients with SCD (n = 4) were cultured as described in Figure 4. Levels of P-SFK Y397 in proliferated B cells and plasma B cells were assessed by flow cytometry. Fold changes in adj MFI were calculated and illustrated in the column plots. ∗*P* < .05; ∗∗*P* < .01. Adj MFI, adjusted mean fluorescence intensities; GCB, germinal center B cells; PBC, plasmablasts; PC, plasma cells.

SS mice were immunized with NP-CGG in the presence or absence of ASNase as described in Figure 3, and SFK activation was assessed in splenic B-cell subsets. ASNase treatment did not alter total expression levels of SFK members Fyn and Lyn (Figure 5A), suggesting that Asn depletion does not affect SFK protein abundance. During activation, autophosphorylation on tyrosine at sites 397 to 421, depending on the specific SFK protein, increases SFK activity. We next evaluated SFK activation using an antibody against phosphorylated SFK (P-SFK Y397). ASNase treatment significantly reduced P-SFK Y397 levels in germinal center B cells, plasmablasts, and plasma cells, but not in resting B cells (rest B; Figure 5B; supplemental Figure 6), consistent with its selective effect on activated B-cell populations. In AA control immunized mice, ASNase treatment had no effect on SFK activation in activated B cells (plasma cells) or resting B cells (supplemental Figure 7), suggesting an SFK-independent inhibition of alloimmunization by ASNase in non-SCD mice.

To determine whether SFK signaling contributes to RBC alloimmunization, SS mice were treated with the SFK inhibitor PP2^36^, ^37^, ^38^ during HuGPA-RBC immunization as described in Figure 3. PP2 treatment (5 mg/kg, IP injection, twice per week, given 2 hours before the first immunization and continued until mice were euthanized) significantly reduced anti–HuGPA-RBC IgG responses (Figure 5C), supporting a role for SFK signaling in promoting humoral immune responses in this model.

To assess whether Asn depletion similarly affects human B cells, we analyzed SFK activation in in vitro–activated B cells from HDs and patients with SCD cultured with ASNase as described in Figure 4. ASNase reduced P-SFK Y397 levels in activated and plasma B cells from patients with SCD, whereas no significant reduction was observed in HD B cells (Figure 5D).

## Discussion

In this study, we reveal a previously unrecognized erythroid cell–metabolism–immune axis that potentially regulates humoral immunity in SS mice. Our data suggest that Mito^+^ circulating SCD erythroid cells can synthesize Asn, contributing to elevated plasma Asn levels and promotion of humoral immune responses in SS mice. Functionally, depletion of Asn using ASNase suppresses plasma cell differentiation and reduces RBC alloimmunization, with a more pronounced effect in SCD. Mechanistically, robust B-cell suppressive effects of ASNase in SCD are associated with inhibition of SFK activation in activated B cells. Together, these findings highlight the therapeutic potential of ASNase and SFK inhibitors for mitigating RBC alloimmunization in SCD.

A key implication of our finding is that circulating erythroid cells, traditionally considered metabolically limited, may actively regulate systemic amino acid availability and downstream immune responses. Although mature RBCs lack organelles, reticulocytes and mitochondria-retaining erythroid cells in SCD exhibit enhanced metabolic capacity. Our data reveal that these cells contribute to increased Asn synthesis through a mitochondrial-dependent pathway, establishing a mechanistic link between erythroid abnormalities and systemic metabolic dysregulation in SCD.

Our findings further suggest that erythropoiesis-associated processes^39^ contribute to immune regulation in SCD. Prior studies have revealed that erythropoietin (EPO) enhances humoral immune responses in mice,^40^ although the mechanisms remain unclear. Our data imply that elevated EPO levels and reticulocytosis in SCD may expand the pool of Mito^+^ erythroid cells, thereby augmenting Asn production. In addition, reticulocyte-rich donor RBCs have been associated with increased alloimmunization, potentially due to enhanced clearance of donor RBCs and cytokine release.^41^ Whether EPO-driven metabolic reprogramming contributes to alloimmunization risk in SCD warrants further investigation.

Although our data support a central role for Asn, contributions from downstream metabolites cannot be excluded. ASNase treatment selectively reduced plasma Asn without inhibiting other measured metabolites (data not revealed), confirming its efficacy and specificity. Interestingly, ASNase treatment increased levels of l-aspartic acid (Asp), N-acetyl-l-aspartic acid (N-acetyl-Asp), and oxidized glutathione (GSSG) in the plasma, whereas reduced glutathione remains unchanged (supplemental Figure 8). The elevation in Asp and N-acetyl-Asp levels is expected, as Asp is a direct product of ASNase activity and N-acetyl-Asp is derived from Asp. However, the mechanism underlying the increase in GSSG remains unclear. Prior studies indicate that Asn supports humoral immunity,^42^^,^^43^ whereas GSSG promotes T helper 1 (Th1)–biased responses in asthma models.^44^ Whether these changes in downstream metabolites contribute to the immunosuppressive effects of ASNase remains to be determined.

We observed elevated Asn levels in erythroid cells from patients with SCD but not consistently in the plasma. This discrepancy may reflect species-specific differences, including the lower frequency of reticulocytes and Mito^+^ mature RBCs in patients (∼5%-10%) compared with SCD mice (∼40%-50%), as well as clinical heterogeneity. Notably, reduced plasma Asn levels have been reported during VOC,^45^, ^46^, ^47^ a state associated with worsened anemia,^48^ which may result in acute reductions in Mito^+^ erythroid cells, accounting for decreased plasma Asn levels during VOC. Such reports further suggest that Asn levels may vary across clinical contexts in SCD. The relatively small size of our human cohort represents a limitation, underscoring the need for larger studies to define the relationship between Asn levels and alloimmunization risk in SCD. More broadly, alterations in circulating Mito^+^ erythroid cells have been reported in other conditions, including anemia,^20^ lupus,^49^ and Rett syndrome,^50^ raising the possibility that erythroid-derived Asn may represent a broader mechanism of immune regulation.

ASNase exerted a more pronounced suppressive effect on B-cell responses in the SCD setting, as demonstrated in both in vivo and in vitro models. Our data suggest that this enhanced efficacy reflects SCD-specific modulation of SFK signaling in activated B cells, which are not observed in non-SCD setting; however, the precise alterations within this pathway remain to be defined. Notably, SFK family members exhibit substantial functional redundancy, allowing compensation within shared signaling networks. As a result, delineation of individual SFK functions typically requires simultaneous deletion of multiple family members^35^; such SFK knockout models, even if available, were not included in this study. SFKs play complex roles in B-cell receptor signaling,^51^^,^^52^ functioning both as positive regulators,^35^^,^^53^^,^^54^ through phosphorylation of immunoreceptor tyrosine-based activation motif (ITAMs), and as negative regulators through phosphorylation of immunoreceptor tyrosine-based inhibitory motifs (ITIMs) and inhibitory sites on Syk.^55^, ^56^, ^57^ Whether the balance between activating and inhibitory SFK signaling is altered in SCD and contributes to ASNase sensitivity warrants further investigation. Notably, our prior work demonstrated that hemolysis inhibits Src-Syk activation in human B cells,^32^ raising the possibility that hemolysis may modulate ASNase responsiveness in SCD. Collectively, these findings support the potential of SFK inhibition as a therapeutic strategy to attenuate humoral immune responses in SCD. Clinically available agents such as nilotinib and dasatinib,^58^, ^59^, ^60^, ^61^, ^62^ originally developed as B-cell receptor–ABL inhibitors, also potently inhibit SFKs and merit further evaluation in this context.

Multiple mechanisms have been implicated in Asn-mediated regulation of immune cell function. Consistent with recent studies, Asn can enhance CD8^+^ T-cell activation by directly binding and activating Lck, an SFK family member.^9^ Additional mechanisms include inhibition of NRF2 signaling.^63^ In contrast, in HD conditions, ASNase suppressed plasma cell differentiation without affecting SFK activation, suggesting an SFK-independent mechanism. Indeed, Asn restriction has been found to impair germinal center formation in mice^22^ and inhibit human B-cell proliferation and antigen presentation function,^64^ potentially through inhibition of mitochondrial function. Whether ASNase-mediated suppression of plasma cell differentiation in HD settings is driven by mitochondrial inhibition warrants further study. Collectively, these findings suggest that Asn regulates adaptive immunity through multiple, context-dependent mechanisms.

Adjuvants increase humoral immune responses through distinct pathways. In our study, CpG (a toll-like receptor 9 agonist that elicits a Th1-biased response) and Alhydrogel (an alum-based adjuvant that drives a Th2-biased immune response) were used in RBC alloimmunization and NP-CGG models, respectively. SS mice exhibited reduced alloimmunization responses under CpG stimulation but comparable responses in the NP-CGG model. This discrepancy may reflect a Th1-biased immune deficiency in SS mice or differences in immunization route (IV for the RBC-alloimmunization model vs IP in the NP-CGG model). Importantly, ASNase consistently suppressed humoral responses in SS mice across both models, indicating that its effects are independent of adjuvant and route of immunization. These data support a model in which ASNase primarily targets plasma cell differentiation. The mechanisms underlying reduced CpG-driven alloimmunization in SS mice remain to be elucidated.

Conflict-of-interest disclosure: The authors declare no competing financial interests.

## Supplementary Material

Supplemental Methods, Figures, and References
