# Supplementary material for: Targeting erythroid cell–derived asparagine inhibits alloimmunization in sickle cell disease
Source: Blood Adv. 2026 Apr 20;10(12):4347–57. doi: 10.1182/bloodadvances.2026020233 (PMC13276564; doi:10.1182/bloodadvances.2026020233)
Supplement: Supplemental Methods, Figures, and References [file BLOODA_ADV-2026-020233-mmc1.pdf]

Materials and Methods:

Human Samples

All studies were approved by the Institutional Review Board of New York Blood Center (NYBC), Montefiore Health Center, and the University of Illinois at Chicago. SCD patient blood samples were obtained after informed consent. For metabolomic analysis, whole blood samples (EDTA as anticoagulant) from SCD patients without transfusion (HgbS%: 79.3±10.8) were collected. For human B cell cultured experiments, our cohort of SCD patients (n=16, median age:20 Years, range:15-39; 50% female, HgbS%: 41.2±18.56,) were on a chronic RBC transfusion therapy (every month for at least two years using leukodepleted units, phenotype matched for C, E and K red cell antigens and without any SCD complications at the time of the blood draw for the study; 56% had a history of at least one RBC alloantibody). Blood samples were processed within 18 hours after collection. As a control, de-identified blood samples were collected on the same day from race-matched healthy blood donors (HDs) from NYBC and processed at the same time as SCD samples. Leukocyte-enriched products from de-identified HDs of NYBC were used for B cell isolation for *in vitro* culture studies.

Mouse strains

All procedures were approved by the Institutional Animal Care and Use Committee of NYBC. Mice were housed in the animal facility of NYBC. Townes sickle mice (B6;129-Hbbtm2(HBG1, HBB∗)Tow /Hbbtm3 (HBG1,HBB)Tow Hbatm1(HBA)Tow/J, Strain #: 013071) were purchased from Jackson Laboratory (Bar Harbor, ME) and bred at the NYBC animal facility. HbSS-Townes mice (referred to as SS mice) have the human α-globin gene and human sickle β^S- and fetal $^A\gamma$-globin gene. HbAA-Townes mice (referred to as AA) have human α-globin gene and linked human β^A- and fetal $^A\gamma$-globins. C57BL/6J mice (C57 mice) were purchased from Jackson Laboratory. HuGPA-Tg mice[1] were generated through transgenic insertion of the human glycophorin A (GPA) gene into FVB background mice and express the human GPA protein (HuGPA) on circulating RBCs. AA and SS mice aged 10 to 16 weeks and C57 mice aged 10-12 weeks were used in the studies. Both male and female mice were used in all experiments. The ages and sex of mice were matched between the groups.

Antibodies and Reagents

Antibodies: anti-human CD71 (PE, Cat. No: 555537, BD Biosciences), anti-human CD41 (PE-Cy7, Cat. No: 555537, Biolegend), anti-human CD45 (BUV395, Cat. No: 563792, BD Biosciences), anti-human CD19 (PE-Cy7, Cat. No: 557835, BD Biosciences), anti-human CD45 (BUV737, Cat. No: 568524, BD Biosciences), anti-human CD27 (BV711, Cat. No: 564893, BD Biosciences), anti-human CD38 (BV605, Cat. No: 740401, BD Biosciences), anti-human IgM (BV786, Cat. No: 740998, BD Biosciences), anti-human IgG (R718, Cat. No: 751917, BD Biosciences), anti-Blimp-1 (BV421, Cat. No: 565276, BD Biosciences), anti-human CD148 (BB700, Cat. No: 745938, BD Biosciences), anti-Phospho-LYN (Tyr397)/LCK (Tyr394)/HCK (Tyr411)/BLK (Tyr389) (Cat. No: 70926S, Cell Signaling Technology, Danvers, Massachusetts, USA) was conjugated with R-Phycoerythrin using Lightning-Link R-PE Antibody Labeling Kit (Cat. No: 703-0010, Novus Biologicals, Oakville, ON L6M 2V5, Canada), PerCP-Cy™5.5 Rat Anti-Mouse Ly-6C (BD Pharmingen™, Cat. No: 560525), Anti-CD267 (TACI) Monoclonal Antibody APC (eBioscience™, Cat. No:17-5942-82), BV421 Rat Anti-Mouse CD138 (BD Horizon™, Cat. No:562610), anti-mouse CD71 (BV605, Cat. No: 563013, BD Biosciences), anti-mouse CD45 (BUV805, Cat. No: 568336, BD Biosciences), anti-mouse CD41 (PE, Cat. No: 558040, BD Biosciences), NP-CGG PE (Biosearch Technologies, Cat. No: N-5070-1). anti-mouse/human CD45R/B220 (Pacific Blue, Clone RA3-6B2, 103227, BD Bioscience), anti-Mouse CD138 (APC, Clone 281-2, 558626, BD Bioscience), Anti-Mouse T- and B-Cell Activation Antigen (FITC, Clone GL7, 553666), Anti-Mouse CD95 (Biotin, Clone Jo2, 554256, BD Bioscience), Streptavidin (PerCP/Cyanine5.5, 405214, BD bioscience).

Other reagents: eBioscience™ Fixable Viability Dye eFluor™ 780 (Cat. No: 65-0865-18, Thermo Fisher Scientific) and eBioscience™ Fixable Viability Dye eFluor™ 506; (Cat. No:65-0866-14, Thermo Fisher Scientific), were used for cell viability analysis.

RBC alloimmunization model

Whole blood collected from HuGPA-Tg mice was passed through an Acrodisc (WBC) filter (AP-4951, Pall Corporation, Puerto Rico, USA) to remove white blood cells. The filtered cells were washed twice

with Dulbecco's Phosphate Buffered Saline (DPBS), followed by centrifugation at 200 × g for 10 minutes at room temperature (brake setting 2) for platelet depletion. HuGPA-RBC pellet was re-suspended with the same volume of DPBS. 100µl freshly prepared HuGPA-RBC suspension was transfused with CpG as adjuvant (ODN 1826 VacciGrade, InvivoGen, San Diego, 50µg/mouse for control mice, 2µg/mouse for sickle mice). One week later, mice were transfused again with the same volume of HuGPA-RBC suspension without CpG. One week after the second immunization, blood samples were collected, and plasma anti-HuGPA-RBC IgG levels were measured. For measuring the anti-HuGPA-RBC IgG levels in the plasma samples, diluted plasma was incubated with HuGPA-RBCs (50µl 1/10000 diluted HuGPA-RBCs in DPBS) at 37 °C for 1 hour. After incubation, HuGPA-RBCs were washed twice with DPBS and stained with anti-mouse IgG antibody (1:100 dilution in 50µl DPBS) at 4 °C for 15 minutes. Cells were then washed twice with DPBS, resuspended in DPBS, and analyzed by flow cytometry. The mean fluorescence intensities of HuGPA-RBCs were used as the indicator for IgG levels in plasma.

Asparaginase (ASNase) treatment: ASNase (Spectrila, European Medicines Agency, 2.5 U/mice) was administered I.V. to mice twice per week, starting 1 week before immunization and continuing until mice were sacrificed.

Asn treatment: C57 mice were treated with Asn (25mg/kg, I.P. injection, Cat. No: B21473.22, Thermo Fisher Scientific) twice per week, starting 1 week before immunization and continuing until mice were sacrificed.

PP2 treatment: PP2 (Catalog#1407, Tocris Bioscience, 5mg/kg) was administered by I.P. injection to mice twice per week, given 2 hours before the first immunization and continued until mice were sacrificed.

NP-CGG immunization

Mice were intraperitoneally injected with NP-CGG (50µg/mouse, N-5005D-5, LGC Biosearch Technologies, Hoddesdon, UK) with Alhydrogen adjuvant (50µl/mouse, Cat. No:vac-alu-50, InvivoGen, San Diego, CA). Plasma and spleen samples were collected one week after immunization. The levels of plasma NP-CGG-specific IgG were analyzed by ELISA. Splenic NP-CGG-specific plasma cell frequency in the total splenic white blood cell population was analyzed by flow cytometry.

ELISA (enzyme-linked immunosorbent assay) for NP-specific immunoglobulins

NP32-BSA (Cat.#: N-5050H-10, LGC Biosearch Technologies) and ELISA buffer kit from Invitrogen (Cat.#: Catalog number CNB0011) were used for the assay following the manufacturer's instructions. Briefly, 50µl NP32-BSA (1 µg/ml) was coated on half-area ELISA plates at 4°C overnight. After 3 washes, 150µl of blocking buffer was added to each well, and the plates were incubated at room temperature for 1 hour. Plasma samples from mice were diluted (1/2000) and, after decanting the blocking buffer, 50µl of diluted serum was distributed into each well. After 1-hour culture at room temperature and 3 washes, polyclonal anti-mouse IgG (1/3000; Invitrogen, Cat. #: G-21040) conjugated to HRP (horseradish peroxidase) was added. After 1-hour incubation at room temperature and 4 washes, 50 µl TMB was added, and the reaction was stopped by 2 M H_2SO_4 (50 µl per well). The optical density (OD) at 450 nm was read in a FLUOstar Omega microplate reader (BMG LABTECH, Cary, NC).

Sample preparations for metabolomic analysis

Leukoreduced mouse and human circulating erythroid cells were prepared as detailed above using Acrodisc WBC Filter. 20µl circulating erythroid cell samples were collected and stored at -80ºC for metabolomic analysis.

Whole blood samples from mice and humans were centrifuged for 10 min (4ºC, 1000G). 20µl plasma samples were collected and stored at -80ºC for metabolomic analysis.

Considering that the volume of erythrocytes is different between erythrocytes from AA vs SS mice and reticulocytes vs mature RBCs, the same volume, instead of the same number, of erythrocytes was used for comparison. A volume of 15 µl of total/CD71+/CD71- erythroid cell pellet (~80X10^6/well) were resuspended in 200 µl of DMEM (Cat. No: 11960044, Thermo Fisher Scientific,) supplemented with 25 mM HEPES (pH 7.5), 1 mM sodium pyruvate, 100 U/mL penicillin-streptomycin, and 4 mM [U-$^{13}C_5$]-L-glutamine (Cat. No: CLM-1822-H-0.5, Cambridge Isotope Laboratories, Tewksbury, MA) and incubated overnight at 37 °C in 5% CO_2 after which culture supernatants were collected, and cells washed twice with DPBS. A 9-hour incubation period was selected based on preliminary optimization

to allow sufficient accumulation of labeled metabolites while minimizing hemolysis of SS erythroid cells during culture. Both the supernatants and cell pellets were subjected to targeted metabolomics analysis. The experiment was performed in triplicate.

Mouse RBC transfusion

RBC sample preparation: whole blood collected from mice was passed through an Acrodisc (WBC) filter to remove white blood cells. The filtered cells were washed twice with DPBS, then centrifuged at 200 × g for 10 minutes at room temperature with slow brake (brake setting 2) to deplete platelets. The RBC pellet was resuspended with the same volume of DPBS for transfusion.

Normal RBC transfusion to SS mice: Purified red blood cells (RBCs) were obtained from C57 mice. C57 mice were used instead of AA mice because the transfusion study required a large number of donor animals (n= 40); AA mice are substantially more expensive, and their RBC lifespan is comparable to that of C57 mice. RBC suspensions were transfused via retro-orbital injection at a volume of 400 μL per injection, administered twice per week, with injections separated by 12 hours, for a total duration of 4 weeks. Blood samples were collected after four weeks of transfusions.

AA and SS RBC transfusion to C57 mice: Purified AA and RBC suspension (Hct 50%) was transfused through retro-orbital injection (400μl/time) on day 1 and day 3. Plasma samples were collected on day 5. Levels of phosphatidylserine (PS) on erythroid cells in SS mice samples were assessed by flow cytometry before and after RBC sample preparation using PE-conjugated Annexin V (Cat. No: 560930, BD Biosciences) and BD Pharmingen™ Annexin V Binding Buffer, 10X concentrate (Cat. No: 556454, BD Biosciences) following the manufacturer's protocol. The frequency of PS-positive erythroid cells was less than 1% before and ~3% after SS erythroid cell sample preparation (Fig.S9).

Mice CD71+/- RBCs isolation

Mouse RBCs were prepared by passing 200μl of whole blood through an Acrodisc WBC Filter (PN: AP-4951, Pall Life Sciences, Port Washington, NY) to remove white blood cells. The filtered blood was washed twice with 2 mL of Dulbecco's Phosphate Buffered Saline (DPBS) by centrifugation at 200 × g for 10 minutes at room temperature (brake setting 2) to remove platelets.

To isolate $CD71^+$ and $CD71^-$ RBCs, 70μl of RBC pellet was resuspended in 2 mL DPBS and incubated with 40μL biotin-labeled anti-mouse CD71 antibody (Cat. No: 557416, BD Biosciences) at 4 °C for 10 minutes with rotation. Next, 200μl of anti-biotin microbeads (Cat. No: 130-090-485, Miltenyi Biotec, Bergisch Gladbach, Germany) were added, and the suspension was rotated for another 15 minutes at 4 °C. Cells were then washed with MACS buffer (DPBS supplemented with 0.5% BSA and 2 mM EDTA) to remove excess antibodies and microbeads. $CD71^+$ and $CD71^-$ RBCs were separated using a QuadroMACS separator (Cat. No: 130-091-051, Miltenyi Biotec), followed by centrifugation at 400 × g for 10 minutes at 4 °C (brake setting 2) to obtain the respective cell pellets.

Targeted metabolomics

The targeted metabolomic analysis was performed at the Weill Cornell Medicine Proteomics and Metabolomics Core Facility (https://research.weill.cornell.edu/core-facilities/proteomics-metabolomics). The metabolite intensities were provided to NYBC for further analysis. Briefly, pre-chilled 80% methanol (-80 °C) were used for extraction. The extract was dried with a Speedvac and redissolved in HPLC-grade water before it was applied to the hydrophilic interaction chromatography LC-MS. Metabolites were measured on a Q Exactive Orbitrap mass spectrometer (Thermo Scientific), which was coupled to a Vanquish UPLC system (Thermo Scientific) via an Ion Max ion source with a HESI II probe (Thermo Scientific). A Sequant ZIC-pHILIC column (2.1 mm i.d. × 150 mm, particle size of 5 μm, Millipore Sigma) was used for separation of metabolites. A 2.1 × 20 mm guard column with the same packing material was used to protect the analytical column. The flow rate was set at 150 μL/min. Buffers consisted of 100% acetonitrile for mobile phase A, and 0.1% NH_4OH/20 mM CH_3COONH_4 in water for mobile phase B. The chromatographic gradient ran from 85% to 30% A in 20 min followed by a wash with 30% A and re-equilibration at 85% A. The Q Exactive was operated in full scan, polarity-switching mode with the following parameters: the spray voltage 3.0 kV, the heated capillary temperature 300 °C, the HESI probe temperature 350 °C, the sheath gas flow 40 units, the auxiliary gas flow 15 units. MS data acquisition was performed in the m/z range of 70–1,000, with 70,000 resolution (at 200 m/z). The AGC target was 1e6 and the maximum injection time was 250 ms. The MS data was processed using XCalibur 4.1

(Thermo Scientific) to obtain the metabolite signal intensities. Identification of metabolites and stable isotopes required exact mass (within 5ppm) and standard retention times.

Human B Cells isolation and cultures

Naïve B cells were isolated as previously described[2]. For asparagine (Asn) or asparaginase (ASNase) treatments, isolated B cells were labeled with CFSE (Cat. No: V12883, Thermo Fisher Scientific) and cultured in U-bottom 96-well plates in the presence of goat anti-human IgA+IgG+IgM (H+L) $F(ab')_2$ fragment (3.25 µg/mL, Cat. No: 109-006-064, Jackson ImmunoResearch), CD154 (200 ng/mL, Cat. No: 6245-CL-050/CF, R&D Systems), CpG (500nM, Cat. No: tlrl-2006-1, InvivoGen), and IL-2 (50 ng/mL, Cat. No: 1081-IL, R&D Systems) at 37 °C in 5% CO_2 for 5 days, after which they were harvested for flow cytometry analysis.

For Asn treatment, 2.5×10^4 naïve B cells were cultured in 200µl RPMI 1640 medium (Cat. No: R9010-01, USBiological Life Sciences) supplemented with 10% dialyzed FBS (Cat. No: 26400044, Thermo Fisher Scientific), 2 g/L D-glucose (Cat. No: A2494001, Thermo Fisher Scientific), 2 mM L-glutamine, 1 mM sodium pyruvate, 100 U/mL penicillin-streptomycin (Cat. No: 15140-122, Thermo Fisher Scientific), 10 mg/L glycine, 200 mg/L L-arginine, 20 mg/L L-aspartic acid, 65 mg/L L-cystine 2HCl, 20 mg/L L-glutamic acid, 15 mg/L L-histidine, 20 mg/L L-hydroxyproline, 50 mg/L L-isoleucine, 50 mg/L L-leucine, 40 mg/L L-lysine hydrochloride, 15 mg/L L-methionine, 15 mg/L L-phenylalanine, 20 mg/L L-proline, 30 mg/L L-serine, 20 mg/L L-threonine, 5 mg/L L-tryptophan, 29 mg/L L-tyrosine disodium salt dihydrate, 20 mg/L L-valine, and varying concentrations of Asn (50, 15, 12.5, 6, and 0 mg/L; Cat. No: B21473.22, Thermo Fisher Scientific).

For ASNase treatment, 5×10^4 naïve B cells were cultured in 200µl RPMI 1640 medium (Thermo Fisher Scientific, Cat. No: 22400105) supplemented with 10% FBS (Cytiva, Cat. No: SH30071.03HI), 1 mM sodium pyruvate, 100 U/mL penicillin-streptomycin, and various concentrations of asparaginase (1, 0.5, 0.25, and 0mU/L, Spectrila, European Medicines Agency).

Flow cytometric analysis

Human B cells: B cells cultured in U-bottom 96-well plates were harvested by centrifugation at 300 × g for 5 minutes at room temperature, followed by one wash with 200µl MACS buffer. Cells were then stained for surface markers and viability using antibodies and reagents diluted in MACS buffer (1:100 anti-CD19, 1:400 anti-CD45, 1:400 anti-CD38, 1:400 anti-CD27, and 1:1000 eBioscience™ Fixable Viability Dye eFluor™ 780) for 30 minutes at 4°C. After staining, cells were washed with 200 µl MACS buffer, then fixed and permeabilized with 150µl eBioscience™ Foxp3/Transcription Factor Fixation/ Permeabilization Concentrate and Diluent (Cat. No: 00-5521-00, ThermoFisher Scientific) for 45 minutes at 4°C. Subsequently, cells were washed twice with Permeabilization Buffer (Cat. No: 00-8333-56, Thermo Fisher Scientific). Intracellular staining was performed in 25µl permeabilization buffer containing diluted antibodies (1:200 anti-IgM, anti-IgG, anti-Blimp-1, and anti-Phospho-LYN (Tyr397)/LCK (Tyr394)/HCK (Tyr411)/BLK (Tyr389)) for 45 minutes at 4°C. After two additional washes with Permeabilization Buffer, cells were resuspended in 70µl DPBS and analyzed using a BD LSRFortessa™ 5 Cell Analyzer (BD Biosciences).

Human or Mouse RBCs: 1µl of whole blood was diluted in 1 ml DPBS. Subsequently, 200µl of the diluted blood suspension was stained with anti-CD45, anti-CD71, and anti-CD41 antibodies (1:200 dilution) at 4 °C for 30 minutes. After staining, RBCs were washed with DPBS and resuspended in a prewarmed (37 °C) staining solution containing 6.25µM MitoTracker® Deep Red FM and 0.1µg/mL Thiazole Orange in DPBS, then incubated at 37 °C for 30 minutes. Cells were then centrifuged to obtain a pellet, the supernatant was aspirated, and RBCs were resuspended in DPBS with 0.1µg/mL Thiazole Orange. Samples were analyzed using a BD FACSymphony A3 Cell Analyzer (BD Biosciences). For reticulocyte analysis in Fig.S1), diluted RBCs were stained with 0.1µg/mL Thiazole Orange at room temperature for 30 minutes and then directly analyzed using the same instrument.

Mouse B cells: Single-cell suspensions were prepared from mouse spleens using gentleMACS™ tissue Dissociators (Miltenyi Biotec) following the protocols provided by the manufacturer. Single-cell suspensions were prepared from mouse bone marrow using the conventional flushing method. RBCs were depleted using BD FACS lysing solution (BD Biosciences). Splenic white blood cells were stained for surface markers and viability using anti-mouse Ly6C antibody (Percp-cy5.5, BD Pharmingen, 1:400),

anti-mouse CD267 (TACI) antibody (APC, eBioscience, 1:400), anti-mouse CD138 antibody (BV421, BD Horizon, 1:200), and Fixable Viability Dye eFluor™ 506 (eBioscience) at 4°C for 30 minutes. For spleen germinal center B cell staining, biotin-labeled anti-CD95 (Jo2) and fluorescein isothiocyanate (FITC)-labeled anti-T- and B-cell activation antigen (GL7) were used (BD Biosciences). After staining, cells were washed with 200µl MACS buffer, then fixed and permeabilized with 150 µl eBioscience™ Foxp3/Transcription Factor Fixation/ Permeabilization Concentrate and Diluent (Cat. No: 00-5521-00, Thermo Fisher Scientific) for 45 minutes at 4°C. Subsequently, cells were washed twice with Permeabilization Buffer (Cat. No: 00-8333-56, Thermo Fisher Scientific). Intracellular staining was performed using NP-CGG PE (Biosearch Technologies) for 45 minutes at 4°C. After two additional washes with Permeabilization Buffer, cells were resuspended in DPBS and analyzed using a BD LSRFortessa™ 5 Cell Analyzer (BD Biosciences).

The flow cytometry data were analyzed using the FlowJo software (BD Life Sciences).

Statistical analysis

Data are represented as mean values± SEM. GraphPad Prism (GraphPad software, La Jolla, CA) was used for statistical analysis and figure presentation. For data sample size >10, the Shapiro–Wilk test was used for the test of normal distribution. For a data sample size <10, visual methods, including the Normal Q–Q plot, were selected to examine the normal distribution. For normally distributed data, the unpaired two-tailed Student's t-test with Welch correction was used to determine statistical significance between two groups, the Mann-Whitney test was used for non-normally distributed data, and p-values <0.05 were considered statistically significant.

Figure S1: The frequency of reticulocytes in blood in PBS-treated SS mice (“PBS-SCD”, n=4), SS mice transfused with RBCs from C57 mice (“Trans-SCD”, n=7), and untreated AA mice (n=3) was assessed by flow cytometry using Thiazole Orange (TO) staining. (A) Pseudocolor flow cytometry plots showing the gating strategy for detecting reticulocytes (using Thiazole orange staining) within the total circulating erythroid cells. (B) The frequencies of reticulocytes in total erythroid cells based on gating in (A) were shown.

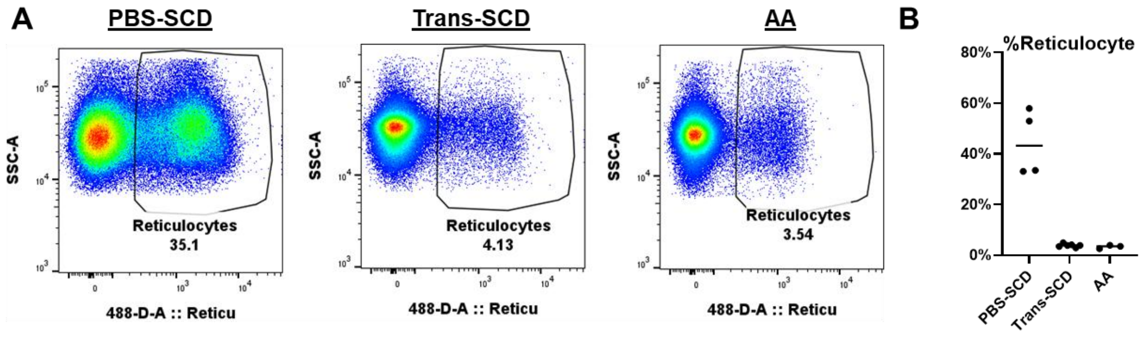

Figure S2: The data were from the same samples used in Figures 2B and 4B. The gating strategies for the analysis of the murine and human total RBC populations are shown. Circulating erythroid cells are referred to as RBCs in the figures and figure legend.

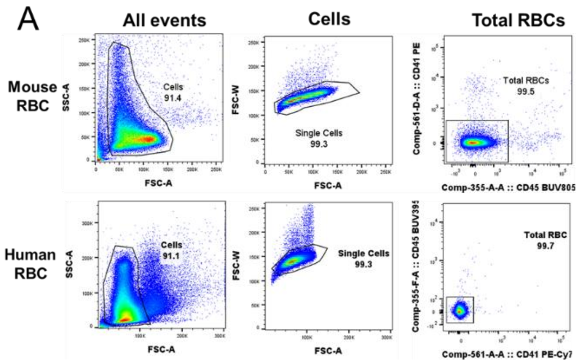

Figure S3: Gating strategy used in flow cytometric analysis of splenic NP+ and NP- plasma cells in NP-CGG immunized mice.

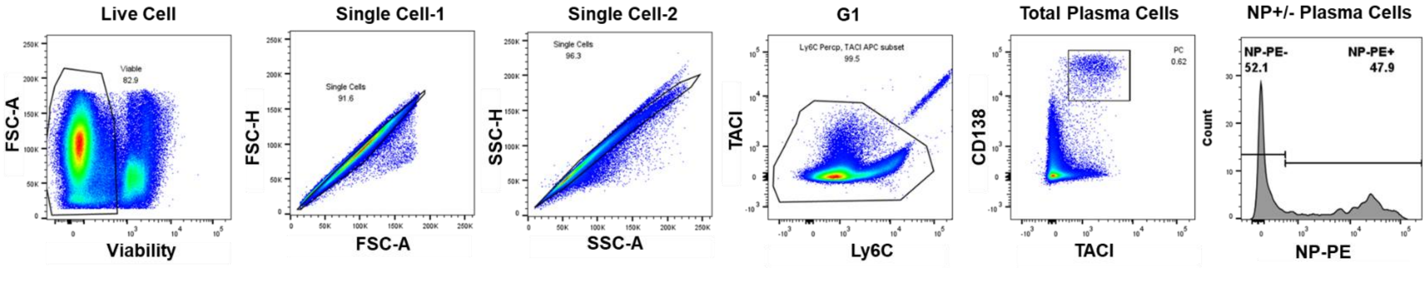

Figure S4: Gating strategy used in flow cytometric analysis of differentiated plasma cells in cultured human B cells.

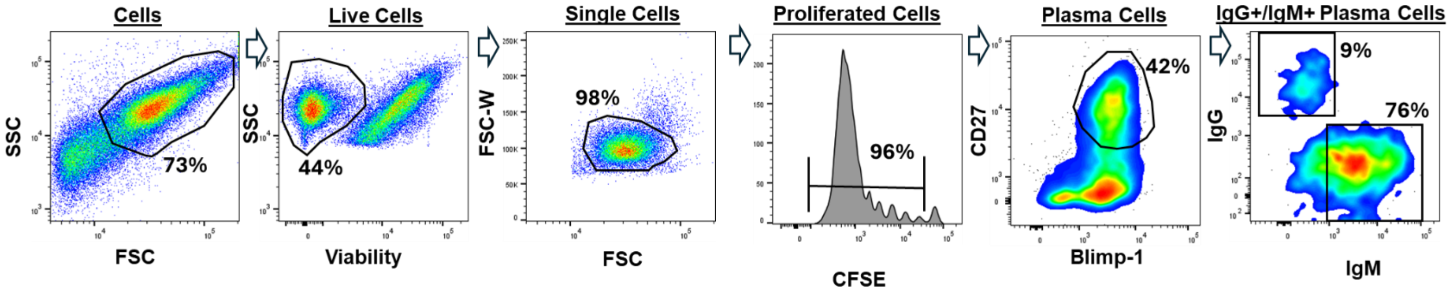

Figure S5: Effect of ASNase on control healthy donor (HD) derived B cell cultures. Human B cells were cultured as described in Fig.4. Graphs showing the effect of increasing concentrations of ASNase on B cell numbers, percentage of live B cells, CFSE MFI of proliferated B cells, and percentage of plasma B cells in divided B cells from individual HDs after culture.

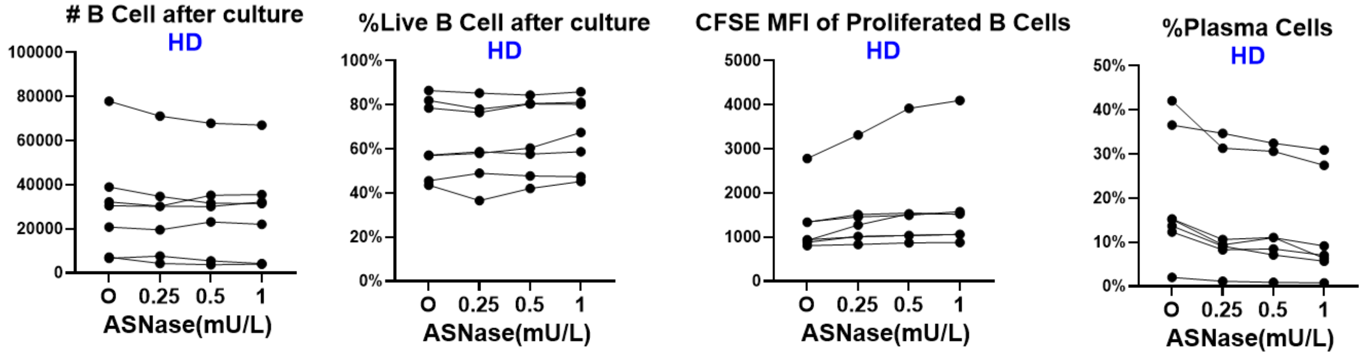

Figure S6: Gating strategy used in flow cytometric analysis of splenic resting B cell (Rest B), germinal center B cell (GCB), plasmablasts (PBC), and plasma cells in (PC) in NP-CGG immunized mice.

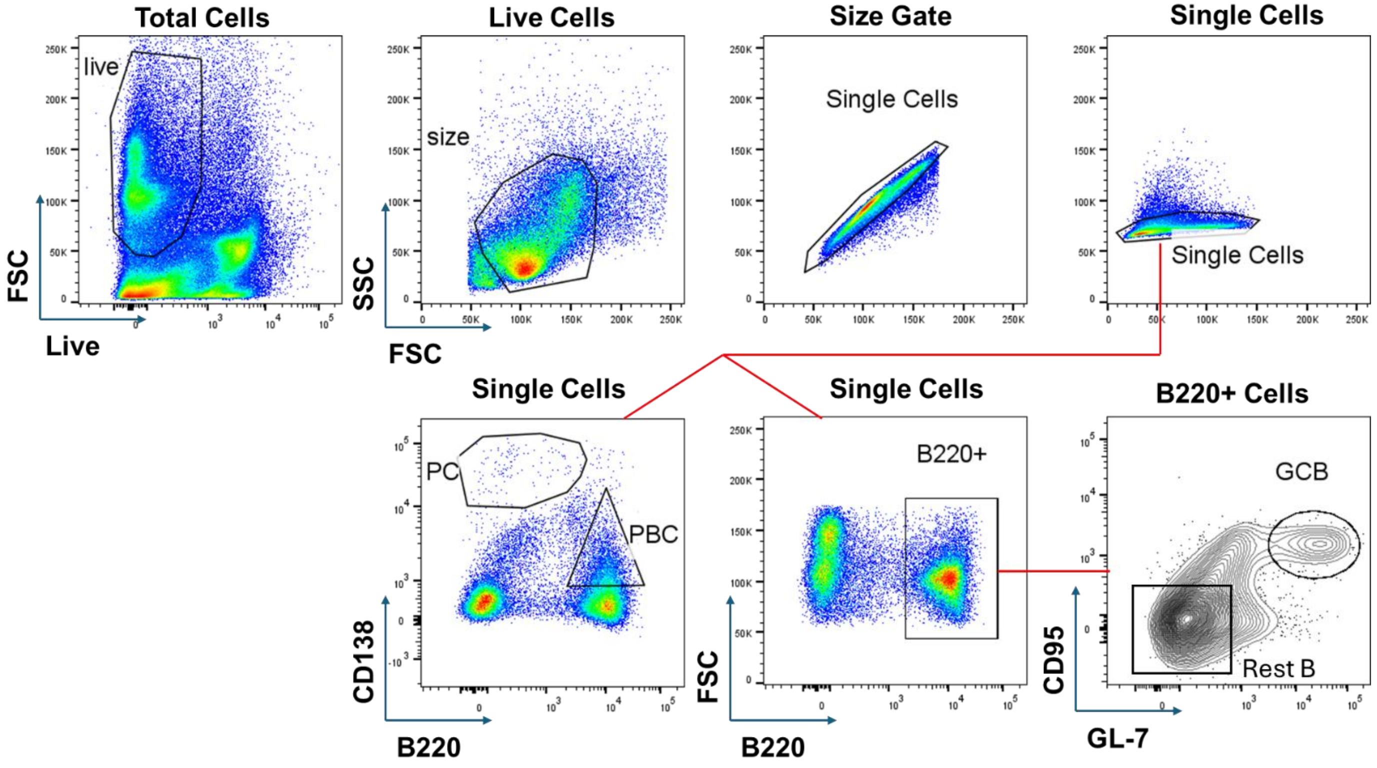

Figure S7: AA mice were immunized with NP-CGG as described in Fig.3 in the presence and absence of ASNase (4 mice/group) (A) Expression levels of Fyn and Lyn in total splenic B cells from immunized mice (4 mice/group) were analyzed by flow cytometry and shown in adj MFI. (B) Levels of P-SFK Y397 in splenic resting B cells (Rest B) and plasma B cells (PC cells) from immunized mice (4 mice/group) were measured by flow cytometry and shown in adj MFI.

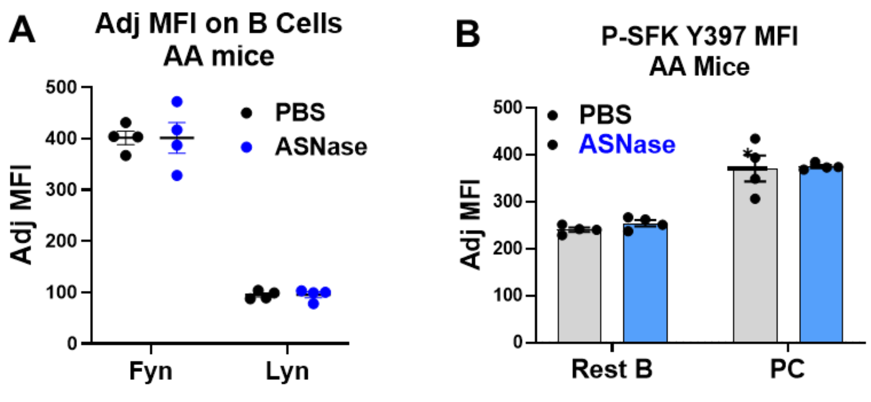

Figure S8: SS mice were treated with ASNase or PBS (3 mice/group) for one week, after which plasma metabolite levels were measured by metabolomic analysis. Three metabolites, including L-Aspartic acid, N-Acetyl-L-aspartic acid, and oxidized glutathione, were significantly increased by 2-fold in SS mice after ASNase treatment.

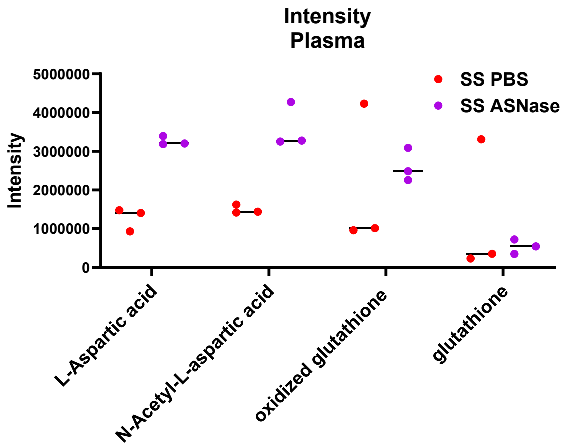

Figure S9: The frequencies of phosphatidylserine (PS) positive circulating erythroid cells were assessed by flow cytometry using PE-conjugated Annexin V.

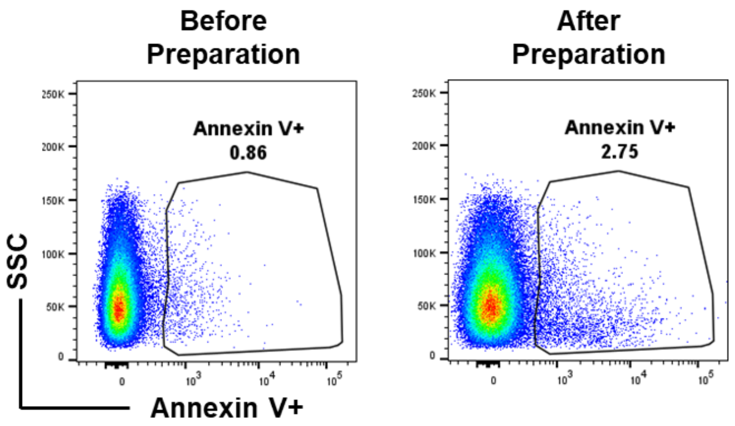

Reference:

1. Auffray I, Marfatia S, de Jong K, et al. Glycophorin A dimerization and band 3 interaction during erythroid membrane biogenesis: in vivo studies in human glycophorin A transgenic mice. *Blood*. 2001;97(9):2872-2878.
2. Pal M, Bao W, Wang R, et al. Hemolysis inhibits humoral B-cell responses and modulates alloimmunization risk in patients with sickle cell disease. *Blood*. 2021;137(2):269-280.
